# Long-Term Followup after Electrocautery Transurethral Resection of the Prostate for Benign Prostatic Hyperplasia

**DOI:** 10.1155/2011/359478

**Published:** 2011-11-28

**Authors:** F. Kallenberg, T. A. Hossack, H. H. Woo

**Affiliations:** ^1^Department of Urology, AMC University, 1100 DD NH Amsterdam, The Netherlands; ^2^Sydney Adventist Hospital, Sydney, NSW 2076, Australia; ^3^Sydney Adventist Hospital Clinical School, University of Sydney, P.O. Box 5017, Wahroonga, NSW 2076, Australia

## Abstract

*Introduction*. For decades, transurethral resection of the prostate (TURP) has been the “gold standard” operation for benign prostatic hyperplasia (BPH) but is based mainly on historic data. The historic data lacks use of validated measures and current TURP differs significantly from that performed 30 years ago. *Methods*. Men who had undergone TURP between 2001 and 2005 were reviewed. International prostate symptom score (IPSS), quality of life (QOL) and peak urinary flow rate (*Q*_max⁡_), and postvoid residual (PVR) were recorded. Operative details and postoperative complications were documented. Patients were then invited to attend for repeat assessment. *Results*. 91 patients participated. Mean follow-up time was 70 months. Mean follow-up results were IPSS—7; QoL—1.5; *Q*_max⁡_—23 mL/s; PVR—45 mL. These were an improvement from baseline of 67%, 63%, 187%, and 80%, respectively. Early complication rates were low, with no blood transfusions, TUR syndrome, or deaths occurring. Urethral stricture rate was higher than anticipated at 14%. *Conclusion*. This study shows modern TURP still produces durable improvement in voiding symptoms which remains comparable with historic studies. This study, however, found a marked drop in early complications but, conversely, a higher than expected incidence of urethral strictures.

## 1. Introduction

Benign prostatic hyperplasia (BPH) is common in men, ranging from 50% in 50 year olds to 90% in 90 year olds. Around 50% of men develop irritating symptoms summarised generally as lower urinary tract symptoms (LUTS). 

For decades, TURP has been the “gold standard” surgical intervention for LUTS associated with BPH. The persistence of traditional diathermy endoscopic prostatectomy as the preferred surgical treatment option for BPH, despite newer technologies constantly being introduced, is based on the belief that outcomes are durable. The evidence that this belief is based on however is now mainly historic [1–4]. 

Although TURP has been around for decades, the current operation differs significantly from that performed 30 years ago. There have been improvements in operative technique, instrument technology, and anaesthetic methods. TURP is now safer, with a much lower mortality rate reported, but the effect these changes have had on long-term outcomes is largely unknown [5, 6]. 

Recent literature on endoscopic, diathermy, prostatectomy focuses either on technical changes or is comparative studies with associated limitations. Recent long-term studies (>3 year) which include TURP use the procedure as the control arm. With these types of studies they typically have small patient groups and adopt strict inclusion criteria (i.e., prostate size restrictions [7–12]) that may render the study sample unrepresentative of the general TURP patient cohort. 

Another limitation of older studies is that many were performed in the 1980's and early 90's [1–5]. These studies typically use retrospective registry data and nonstandardised survey instruments. The IPSS and IIEF only emerged in the late 1990's. This makes comparison with current data difficult. Updated data is needed. 

The aim of this study therefore was to assess the long-term clinical outcomes following TURP in average Australian males.

## 2. Methods

Human Research Ethics Committee (HREC) approval was gained to allow access to men who had undergone TURP between 2001 and 2005 by a single senior urological surgeon. Patients were identified from the practice computer database. 

All eligible files were reviewed. Patients who had or subsequently developed prostate cancer, invasive urothelial carcinoma, or neurogenic bladders were excluded. Men who had undergone previous prostate- or urethra-related surgery were also excluded. 

Eligible patients were posted an HREC-approved participant information letter inviting them for further assessment. If failing to respond, a second letter was sent after checking addresses with their general practitioner. A second failure to respond was deemed a refusal to participate. Subjects that were willing to participate had their files reviewed and baseline data collected. 

Files were initially read to identify eligible subjects. When a subject was deemed eligible their notes were reviewed for baseline data. Baseline data included preoperative international prostate symptom score (IPSS), quality of life (QOL) score, and international index of erectile function (IIEF-5). Peak urinary flow rate (*Q*_max⁡_), voided volume (VV), and postvoid residual (PVR) were documented except for patients in acute urinary retention. If available, prostate-specific antigen (PSA) within one year before surgery was also recorded.

Operation reports were read to confirm operation date and that standard operating procedure had been followed with no other intervention, other than insertion of suprapubic catheter (SPC), being conducted. Prostate tissue resection weight was documented. Postoperative incidence of blood transfusion, TUR syndrome, and death were also documented. 

Patients were then invited to attend an interview, and repeat assessment was conducted. Patients were asked specifically about any urinary tract intervention since their operation, and if any had occurred, they were classified as treatment failure and excluded from repeat assessment. Repeat assessment involved completing the IPSS, QoL, IIEF-5, and patient global impression of improvement score (PGI-I) questionnaires. Sexually active patients were questioned on the presence or absence of antegrade seminal emission with ejaculation. *Q*_max⁡_, VV, and PVR were measured. A voided volume of at least 125 mL was required before a flow rate was considered valid. PVR was assessed using an ultrasound bladder scanner. 

When possible, data was analysed as paired data. Men who were in acute urinary retention before the operation were excluded from paired data analysis. Statistical analysis was performed using Wilcoxon signed ranks test for paired analyses. Two-tailed statistical significance was considered at the 5% level. SPSS statistical software program was used. 

## 3. Results

210 patients undertook TURP between 2001 and 2005. 24 had prior prostate surgery, 38 prostate cancer, and 3 invasive bladder cancer resulting in 65 patients being excluded.

Another 19 patients had died (causes unrelated to the prostate surgery), 24 lost to follow up, 5 unwilling participants, and 6 lived too far away. This left 91 patients available for long-term outcome review. 

Of the 91 men who underwent long-term review, 19 men were deemed treatment failures. Three men had more than one complication; thus, there were 13 urethral strictures; 2 bladder neck contractures, 4 recurrent BPH, and three detrusor failures (Table 1). This left 72 men providing long-term TURP outcome data.

The average age at operation was 67 years and at followup was 72 years (range 57–94 yr). Mean follow-up time was 70 months (range 36–98 months). Twenty men (22%) were in acute urinary retention before the operation.

Baseline parameters were not available in all areas for all 72 men. Voiding parameters were also not available for men in acute retention. Of the available data, the mean baseline results and number of men which contributed to it are given in Table 2. The mean results were IPSS 21; QoL 4; *Q*_max⁡_ 8 mL/s; VV 247 mL; PVR 205 mL; PSA 6.5. 

Most patients had undergone TURP using monopolar diathermy and a 26F continuous flow resectoscope. If prolonged resection time had been anticipated (i.e., large or inflamed prostate), a bipolar Gyrus diathermy device had been used. 

Clopidogrel and/or warfarin (but not aspirin) had been ceased before surgery. Operations were performed either under general anaesthesia or spinal anaesthesia. 1.5% glycine was used for irrigation unless the Gyrus diathermy was used, in which case normal saline was the irrigation fluid. All patients received antibiotic cover on induction. All patients had an irrigation catheter inserted and continuous bladder irrigation (CBI) commenced. In those considered high risk of detrusor failure, a suprapubic catheter had also been inserted. Mean resection weight was 26 g (range 6–75 g). 

Overall at long-term review the mean follow-up IPSS was 7. This is an improvement from baseline of 14 points (Table 3). Paired results were available for 39 men. For these, IPSS changed from 21 (±6.0) at baseline to 7 (±6.5) at followup—an improvement of 67% (Table 4). This is significant under the Wilcoxon's signed rank test (*P* < 0.001). 

Quality of life was improved from baseline, with the average follow-up QoL score being 1.5 (Table 3). 39 men provided paired QoL scores. For them, there was a significant improvement from the initial preoperative 4 (±1.2) to 1.5 (±1.4) (*P* < 0.001) (Table 4). 

The PGI-I question was answered by 67 patients. The mean score was 0.9 (±1.3), and none rated their urinary tract condition as unchanged or worse. The median response was 0. 

Initial baseline voiding parameters showed a mean flow rate of 8 mL/s with a voided volume of 247 mL and postvoid residual of 205 mL. A valid follow-up measurement was available for 43 men. For these men, the mean *Q*_max⁡_ was 22 mL/s (±7.6 mL/s). VV was 424 mL, and PVR was 65 mL (Table 3). 

Paired data was available for 27 men. Significant improvement was recorded in all three parameters at followup. For the paired data, the average flow rate at followup was 23 mL/s, much improved over the initial 8 mL/s (*P* < 0.001) (Table 3). This improvement in flow rate of 15 mL/s equates to an almost threefold increase. 

For these 27 men, the voided volume also remained improved with a mean of 421 mL (±180.6 mL) at review compared to the baseline of 249 mL (Table 4). This is a 69% improvement. Improvement in PVR was also observed with an overall long-term mean value of 45 mL, significantly better than the mean preoperative PVR of 220 mL (*P* < 0.001).

Of the 72 long-term follow-up patients, 43 of them were asked about sexual function. Of these 43 men, 13 (30%) stated they were not sexually active. Of the remaining 30 patients, who regarded themselves as active, the mean IIEF-5 score was 15 (±7). No patient had completed a preoperative IIEF-5 questionnaire. Absence of seminal emission on orgasm was reported in 26 (87%) of these 30 men, while 2 (7%) maintained antegrade ejaculation and another 2 were unsure. 

## 4. Discussion

In this study, of the potential 210 patients who underwent TURP over the 6 year period, 91 patients participated in long-term follow-up assessment. 119 men were excluded: 24 were not primary TURPs, 41 had a urological cancer, 19 had died, 24 were lost to followup, and 11 were unwilling or unable to participate. During this period of time examined, no competing form of BPH surgery was offered, including any clinical trials of new technology. With the introduction of photoselective vaporisation of the prostate (PVP) into this urological practice in 2005, TURP was no longer routinely offered. 

The average follow-up time was just under 6 years (70 months) with a range of 3–8 years. Of the 91 men participating, 19 were deemed treatment failures requiring reintervention. This left 72 men providing long-term outcome data. 

Follow-up parameters included both subjective outcomes assessed by validated questionnaires and objective flowmetry data with voided volume and postvoid residual. When possible, paired data was analysed. 

This study found that IPSS was improved on the long term. The average decrease in IPSS was 14 points, representing a 67% reduction in symptoms. The majority of men, at followup, regarded their urinary symptoms as being mild (IPSS score 7–9). This compared to preoperative assessment where the majority reported their symptoms as severe (IPSS 20–35). This improvement in IPSS is similar to other reports which give a score between 6 and 7.7 after 3–7-year followup [8, 13–16]. These studies, however, used TURP as a control arm in a comparison study rather than looking at the “average” TURP patient as this study has done. 

Not surprisingly, the improvement in lower urinary tract symptoms is mirrored in the quality of life and patient global impression of improvement scores. Most men initially reported they were “mostly dissatisfied” (QoL score of 4) with their symptoms but this improved, and, at followup, they regarded themselves as “pleased” to “mostly satisfied” (QoL score of 1-2). The PGI-1 score, which asked men to compare their current situation to that before the operation, had most men answering “very much better.” This correlated with a median score of zero. No man recorded an unchanged or worse score. 

Long-term improvement in flow rate, voided volume, and postvoid residual was also found in this study. Paired data showed an average postoperative flow rate of 23 mL/s, with an average increase in flow rate of 15 mL/s. This increase in flow rate is one of the highest reported with the average expected improvement in flow rate typically around 10 mL/s [17]. Voided volume and postvoid residual were also improved by 69% and 80%, respectively. 

At review, 43 men were asked about their sexual function. Interestingly, 30% of men stated they were not sexually active and so did not complete the IIEF-5 questionnaire. Of the 30 men who did complete this questionnaire, the average score was 15. Unfortunately no baseline data was available to allow postoperative changes to be calculated. This result, however, can be compared to the general population. Several studies have demonstrated that up to 80% of men over 70 years of age have a degree of sexual dysfunction [18, 19]. While the data in this series is limited, it does suggest that TURP has had no long-term effect on erectile function. It does, however, have a significant impact on antegrade emission. 87% of sexually active men reported loss of emission on orgasm at followup. This finding is similar to most other reports. 

19 (21%) of the 91 men eligible for review were regarded as treatment failures. Of these 19 men, only four (4%) had recurrent BPH as the cause of their recurrent symptoms. The remaining 15 men required intervention for other causes: 14% for urethral strictures and 2% for bladder neck contractors. While this reintervention rate appears high, it is important to note that this figure may be artificially high. 145 men were initially eligible to participate in this study but were excluded due to death (19), lost to follow up (24), or unwilling/able to participate (11). The reintervention rate therefore is somewhere between 13–21%. 

An important clinical distinction exists between reintervention due to prostatic regrowth and due to other complications. This study found that the rate of prostatic regrowth requiring redoing TURP was low, at only 4%, which equates to 0.5% per year. This result is on the lower end of results quoted in the literature which range from 3–15% at 5 years [6]. This is most likely a reflection on the volume of tissue resected, which for this study was 26 g. This resection volume is consistent with other published series with an average resection volume of 15–29 g [5, 20–23]. 

The reintervention rate for urethral strictures identified in this study is somewhere between 9–14%. Compared to the incidence of strictures quoted in the literature (2–10% [6]), this rate is high. The development of a urethral stricture is most likely secondary to instrumentation, technique, or postoperative catheterisation. In this study, all operations were conducted using a 26F continuous resectoscope. The advantages of using a larger, continuous flow, resection sheath are improved irrigation and vision with lower irrigation pressures. This contributes to better haemostasis hence the absence of blood transfusion and the absence of TUR syndrome observed in this study. The use of this large sheath is likely to have helped contribute to the higher resection volume identified in this study. A higher stricture rate associated with larger resection sheaths has however previously been reported [24, 25], and this appears to hold true for this series. Identification of this increased risk however does not translate into the need to abandon their use. Suggestion has been made that routine urethral dilation prior to insertion of the resection sheath may reduce this incidence. Other factors have also been suggested in the literature as contributing to an increased incidence of urethral strictures. These include a high cutting current and use of lubrication [6]. Lowering the first and increasing the second are thought to help minimise stricture rate. The degree to which each of these factors influences the stricture rate is unknown, but most suggestions can be easily implemented with minimal imposition or side effects. Repeat long-term review is then warranted to clarify if these factors help reduce this complication rate. As this was a retrospective analysis, the high stricture rate was not previously recognised and able to be addressed prior to the introduction of PVP which replaced the use of TURP in this urological practice.

There are several short comings with this study. Firstly, this study only compared a subject's voiding symptoms at two points, baseline, and followup, this prevents any attempt to predict an expected projectory. Currently, the literature also does not help in predicting very long-term results. On one hand, a slow decline may be expected. When looking at intermediate (~3 year) studies and comparing them to long-term (>4 year) studies, an increase in IPSS score is observed, with intermediate studies having an IPSS range of 3 to 10 [8, 16, 26], while longer-term studies have a range of 6 to 11 [15, 27, 28]. On the other hand, other studies like Mishriki et al. found the mean IPSS at 6 years was 10 but this improved to 8.5 at 12 years [29]. It is important to remember, however, that changes in voiding symptoms overtime may be better explained in terms of the normal aging process rather than a function of their prostatic disease or an effect of the surgery [12]. 

Another short coming of this study is that it is a retrospective study incorporating only a single surgeon's results. This limitation is minimised by comparing these results to those currently published. 

Despite these shortcomings, an overall durable significant improvement in subjective as well as objective parameters has been shown after a long-term followup of TURP. These results contribute to the objective of setting a standard that other interventions must meet before an alternative “gold standard” for symptomatic benign prostatic hyperplasia can be introduced. However, differences between studies in patient numbers, statistical analyses, inclusion, and exclusion criteria and follow-up periods have shown that a clear protocol should be made for similar follow-up studies in order to make good comparisons possible. In our opinion, the PGI-I question should be included as one of those standards. In order to avoid timeframe-based differences, only recent studies should be used in assessing the long-term followup of TURP.

## Figures and Tables

**Table 1:** Treatment failure post-TURP.

Reason for failure	Number
Urethral strictures	13
Bladder neck contractures	2
Prostatic regrowth	4
Detrusor failure	3

Total*	19

*Three patients had two complications.

**Table 2:** Baseline characteristics for men undergoing TURP.

Parameter	Value
Age	
Number	91
Mean	67
Range	50–88

Questionnaires	
IPSS	
Number	61
Mean	21
Range	5–35
QoL	
Number	64
Mean	4
Range	3–6

Flowmetry	
*Q*_max⁡_	
Number	58
Mean (mL/s)	8
Range	3–18
VV	
Number	57
Mean (mL)	247
Range	125–576
PVR	
Number	49
Mean (mL)	205
Range	15–720

Prostate measure	
PSA	
Number	62
Mean	6.5
Range	0.6–29
Resection weight	
Number	72
Mean	26
Range	6–75

**Table 3:** Overall long-term outcome results post-TURP.

Parameter	Initial	Followup	Difference	% Improvement
IPSS	21	7	14	67%
QoL	4	1.5	2.5	63%
PGI-I		0.9		
*Q*_max⁡_	8	22	14	175%
VV	247	424	177	72%
PVR	205	65	140	68%

**Table 4:** Paired long-term outcome results post-TURP.

Parameter	*n*	Initial	Followup	Difference (%)	*P*
IPSS	39	21	7	14 (67%)	<0.001
QoL	39	4	1.5	2.5 (63%)	<0.001
*Q*_max⁡_	27	8	23	15 (187%)	<0.001
VV	27	249	421	172 (69%)	<0.001
PVR	25	220	45	175 (80%)	<0.001
